# BCC or not: Sufu keeps it in check

**DOI:** 10.18632/oncoscience.134

**Published:** 2015-02-20

**Authors:** Wen-Chi Yin, Zhu Juan Li, Chi-chung Hui

**Affiliations:** Developmental and Stem Cell Biology, Hospital for Sick Children, Toronto, Canada

Basal cell carcinoma (BCC), driven by aberrantly activated HEDGEHOG (HH) pathway, is the most common human malignancy. Current FDA-approved targeted therapy uses Vismodegib to inhibit SMO, a membrane component of the HH pathway. Despite initial impressive tumor regression, the positive clinical response is short-lived in some BCC patients as acquired SMO mutations confer secondary resistance[1]. Clearly, a deeper understanding of the molecular events underlying BCC tumorigenesis is required to devise effective treatments.

The activity of SMO is repressed by the HH receptor PTCH1. Upon HH binding, SMO promotes dissociation of GLI transcription factors from the key negative intracellular regulator SUFU, thereby allowing expression of HH target genes[2]. Mutations in *PTCH1*, *SMO*, and *SUFU*, believed to unleash GLI activity, are frequently found in BCC. SUFU, like PTCH1, is a major negative regulator of the HH pathway. We have previously shown that loss of *Sufu* in mouse keratinocytes promotes Gli2 nuclear localization due to lack of cytoplasmic sequestration, and consequently leads to elevated target gene expression[3]. Surprisingly, unlike *Ptch1*, inactivation of *Sufu* alone in the mouse skin does not cause BCC.

To identify the key oncogenic events in BCC formation, we performed microarray coupled with Gene Set Enrichment Analysis on *Ptch1* and *Sufu* mutants[4]. The comparative analysis revealed that loss of *Ptch1* in keratinocytes led to significant enrichment of gene sets involved in TGF-β signaling and extracellular matrix remodelling, consistent with the tumorigenic phenotype. In contrast, the majority of gene sets uniquely enriched in *Sufu* knockout keratinocytes are involved in cell cycle control, suggesting a novel role of Sufu in cell cycle regulation. Intriguingly, unlike *Ptch1* knockout skin, which showed elevated number of mitotic cells, *Sufu* knockout skin exhibited normal mitotic count. Furthermore, while DNA damage was found in both mutants, *Sufu* knockout cells displayed DNA damage-induced G_2_/M checkpoint cell cycle arrest. These results indicate that *Ptch1* knockout cells are able to override the checkpoint and continue proliferation with the unstable genome while *Sufu* knockouts halt, a key feature likely contributing to their differential cancer phenotypes. Arrest at G_2_ is typically coupled with accumulation of p53, which activates p21 and 14-3-3σ to sequester mitosis-promoting complex Cyclin-B1/CDK1. Strikingly, p53 protein and p21 transcripts remained low in *Sufu* mutants despite the arrest. These findings suggest that while both loss of *Sufu* and *Ptch1* result in increased entry into cell cycle and impairment in p53 response to cell cycle-driven DNA damage, *Sufu* itself may be a positive regulator of cell cycle progression independent of the p53 checkpoint.

Upregulation of the major HH pathway effector, Gli2, is a hallmark of BCC and is observed in *Ptch1* mouse models. Consistent with our finding that loss of *Ptch1* leads to genome instability and evasion of cell cycle checkpoints, Pantazi *et al*.[5] recently demonstrated that overexpression of GLI2 activator (GLI2ßN) in human keratinocytes is sufficient to induce chromosomal aberrations. They also found that GLI2ßN overexpression results in suppression of cell cycle regulators p21 and 14-3-3σ, and induction of anti-apoptotic mechanisms. These lines of evidence suggest that GLI2 is likely the major mediator of the malignant transformation induced by the loss of *PTCH1* during BCC tumorigenesis.

*In vitro* studies demonstrated that HH signaling can positively regulate cell cycle by promoting the expression of cell cycle regulators (D-type cyclins) and preventing the accumulation of p53. These are consistent with the active mitosis and evasion of cell cycle arrest observed in *Ptch1* knockout cells. Our findings suggest that Sufu may also regulate cell cycle. However, it remains unclear why and how the loss of this negative HH pathway regulator causes cell cycle arrest. One possible mechanism is through DNA damage response, which involves the ATM/ATR, CHK1/CHK2, and CDC25C axis to inactivate the Cyclin-B1/CDK1 complex, leading to G_2_ arrest.

**Figure 1:**
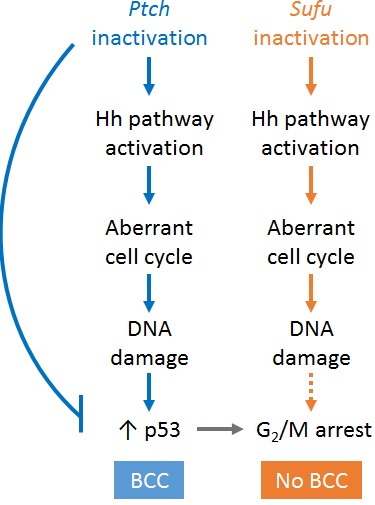
Inactivation of Ptch1 and Sufu lead to distinct cellular events in keratinocytes

Whether Sufu's cell cycle function is Gli-dependent is also unknown. Although ectopic HH target gene expression was found in both *Sufu* and *Ptch1* mutants, Gli2 protein is significantly reduced in *Sufu* mutants compared to wildtype, with exclusive nuclear localization. It is possible that a certain threshold of Gli2 activity is required for evasion of cell cycle arrest and tumor surveillance, and that BCC tumorigenesis is stunted in *Sufu* mutants since the threshold is not achieved.

Double knockout of *Sufu* and *Ptch1* may help determine whether Sufu is required for the rapid cell cycle progression induced by loss of *Ptch1*. In addition, with the recent advances in BioID mass spectrometry[6], identification of Sufu's interactome in keratinocytes may give mechanistic insights into Sufu's involvement in cell cycle regulation. In conclusion, this comparative study of *Ptch1* and *Sufu* mutant mice advanced our understanding of BCC tumorigenesis. Further investigations elucidating the role of Sufu in the cell cycle are warranted for the reason that if Sufu can also function as a positive regulator of the HH pathway, it may represent a potential target for therapeutic intervention of BCC.

## References

[1] Atwood SX (2014). Cold Spring Harb Perspect Med.

[2] Hui CC (2011). Annu Rev Cell Dev Biol.

[3] Li ZJ (2012). Development.

[4] Li ZJ (2014). Oncogene.

[5] Pantazi E (2014). Cell Death Dis.

[6] Roux (2012). J Cell Bio.

